# Academy of Stomatology

**Published:** 1902-09

**Authors:** 

**Affiliations:** Academy of Stomatology


### ACADEMY OF STOMATOLOGY.

A REGULAR meeting of the Academy of Stomatology of Philadelphia was held at its rooms, 1731 Chestnut Street, on the evening of Tuesday, January 28, 1902, the President, Dr. S. B. Luckie, in the chair.

The evening was occupied by the discussion of "Incidents of Office Practice."

### DISCUSSION.

*Dr. E. T. Darby.*—I would like to get the experience of the members here in present methods of obtunding hypersensitivity of the dentine, and of extirpation of the pulp without the use of arsenous acid.

It may be remembered that about a year ago I spoke of a method of injecting carbolic acid and chloroform (equal parts)

into the pulp and extirpating it immediately. I have modified my procedure, and have substituted cocaine crystals, moistened with a drop of water or five per cent. formalin. This is pressed against the pulp with vulcanizable rubber and immediately thereafter the pulp is extirpated, usually within two minutes after pressure is begun, and in almost every instance without pain. What little hemorrhage there has been I have been able to control with chloride of zinc. I have been able to fill the pulp-canal immediately, go on with the operation, and have had no subsequent pericementitis. I can do this in ninety per cent. of exposures in the teeth anterior to the molars. I have learned that in all root-canals the best after-results have been obtained where the canals have been largely reamed to remove the ends of the fibrillæ, thus furnishing a clean open root and lessening the amount of putrescent material. I use a five- or a three-sided reamer, like a Talbot's reamer. My results justify me in continuing that treatment in probably sixty-five per cent. of the exposures that present, and in using just as little arsenic in the devitalization and extirpation of pulps as possible.

*Dr. H. Roberts.*—I would like to endorse Dr. Darby's use of cocaine and pressure. It has been a question with me whether, after the pressure has been exerted and the pulp thoroughly anæsthetized so that it may be removed, its sensibility can or will return. In only one case in my practice have I been able to determine this. I used the method in the buccal cavity of a lower third molar. Containing a large gutta-percha filling on its mesi-occlusal surface. After anæsthetization I took out all the bulb of the pulp and then removed this gutta-percha filling. I expected to have no trouble, for I never had had any, but I could not enter the roots at all; neither could I reproduce the anæsthetic effect of the cocaine and pressure. I had then to resort to arsenic. My failures have been almost invariably in the third molars, where there have been acute symptoms of pulpitis or hyperæmia, so that the pulp cannot take up the drug. One cannot take out such pulps except with much pain to the patient, and expenditure of time. It may be partly anæsthetized, a little of it removed, and the process repeated, and so on, but it is very slow work. We have the same difficulty with arsenic in these cases. Where there has been recent exposure, or where the tooth has not been giving much trouble, I think there is nothing equal to cocaine and pressure. I had another failure yesterday. The patient had complained of odontalgia, and I found an exposed pulp. The pressure produced no more effect on it than to enable me to make a better exposure. I could not repeat the anæsthetic effect. I filled it in with carbolic acid with a tinge of iodine in it, to see if it would stay comfortable and take on a healthy condition, so that I might try the process over another day, which I did. Because of the sensitivity I believe it to be a case of pulp-nodules, as the dentine appears very dense. I made an application of arsenic, and I shall see it again. I have used very little arsenic of late, and only in those cases where there has previously been inflammation of the pulp.

*Dr. Joseph Head.*—Recently I had two teeth partly broken off so that the pulps had to be destroyed. I drilled a small hole, and placed in it a small crystal of cocaine, that could not have been over a fortieth of a grain, and added a droplet of water. With the unvulcanized rubber on the tip of my finger I made pressure, which the patient hardly felt. I was about to drive the liquid farther up into the pulp, when I found, to my surprise, that it was absolutely anæsthetized. It occurred to me that this was due to the fact that I had partly injected the concentrated cocaine into the bulb of the pulp, and the circulation of the pulp had done the rest. This may explain the interesting fact described by Dr. Roberts, that some pulps are not capable of being anæsthetized by this method.

I was also very much interested in Dr. Darby's point of reaming out the pulp-canal. It seems to me an excellent hygienic plan. In the case of neurotic patients there is a tendency to pericementitis that follows the root-filling. I have found that when this is to be feared it is wise, instead of filling immediately, to defer the filling of the canal to the second visit, and after the pulp has been removed to place cotton, well soaked in carbolic acid, in the canal, and while the stump of pulp is under the influence of cocaine drive the cotton with the carbolic acid directly onto the stump and sear it. After this is done the patient can go away, and at his next visit it will be found that the canal is quite insensitive at the apex. Then, to avoid any putrescence, I fill the apical portion of the canal with mummifying paste for a distance of one-eighth inch, then complete in the ordinary way. With these cases I have had excellent results.

*Dr. Robert Huey.*—Many of us find, where there is irritation of the pulp and congestion, that it will never absorb arsenic or cocaine. Under these circumstances, I apply a dressing of morphine and carbolic acid for two or three days, and find that the irritation has been sufficiently reduced to absorb anything without any undue pain. When a pulp is aching I never think of applying arsenic. Thirty-five years ago Dr. J. D. White, in addressing the students, gave us several points in the use of arsenic that I have never forgotten. "Never apply it at night," he said. I believe that is a very good idea. I apply arsenic as early in the day as possible, so that the pulp will be benumbed before lying down at night. He also said, "Put a little arsenic *into* the pulp."

*Dr. Roberts.*—I have used cocaine after arsenic and removed the pulp, though it was not dead from the effects of the arsenic. In one failure that I had with cocaine I had every reason to believe that I would have success, but I could produce absolutely no effect. After wasting considerable time I made an application of arsenic. Two days afterwards I went into the tooth, and to my surprise there was no trace of the pulp. I do not know what caused the pain.

*Dr. Darby.*—There is one point that Dr. Huey touched upon in connection with the use of arsenic that may not be new to the members, and yet it may be to some of them, that is, the application of arsenic at some point remote from the point of exposure. I learned many years ago that when a pulp was exposed an application of arsenic would often keep a patient awake all night. I learned that arsenic could be applied to a point remote from the exposure and cause no pain. Where I find a congested pulp and intend to apply arsenic, I put a sedative dressing of carbolic acid and morphine on the pulp and cover it over without pressure, and then drill into the occlusal or buccal surface and place an application of arsenic in the pit. The morphine paste reduces the congestion, while the arsenic devitalizes by way of the fibrillæ, and usually without pain.

*Dr. Roberts.*—I do not know how long Dr. Darby has been making that sort of an application of arsenic, but I have used it for a long time. Twenty-one years ago a patient came into my hands who had nearly every tooth in his head killed by having arsenic put into the cavity to reduce hypersensitivity. I came to the conclusion that if it would kill pulps through the fibrillæ when you did not wish it to, it would act as well when used intentionally. I have been using that method when the tooth was sensitive and I feared pain from the application.

*Dr. J. T. Lippincott.*—The question suggests itself, if the effect of the arsenic as placed under the enamel, as Dr. Darby mentions, is so far-reaching as to devitalize the pulp, why do we not have an irritation of the peridental membrane from the application of arsenic applied to the pulp?

*Dr. Darby.*—It depends on the quantity, and sometimes it will devitalize the pulp if you put in a little arsenic at that point.

*Dr. A. N. Gaylord.*—I have not been altogether successful with the pressure method, but in nearly every case I have been enabled by it to anæsthetize the surface deeply enough to admit the insertion of the hypodermic needle, by which the remainder of the pulp is easily rendered insensible. It will be found that the pulp is injected with much less pressure than other tissues.

The hypodermic point should be cut off square, with edges bevelled, making it as sharp as possible. This will enable the embedding of the point with much less depth of insertion than would be required with the ordinary long bevelled point.

To avoid hemorrhage, I employ, in the cocaine solution, adrenalin. Whether this agent is familiar to you I know not; it is the active principal of adrenal capsule, and it is a powerful vasomotor constrictor. By its use hemorrhage is almost done away with. I also find it of great value in cocaine solution, where injections are made for the preparation of roots for crowns, there being much less bleeding of the gums.

There is another use of cocaine injection with the hypodermic needle. I have had several occasions where the removal of the pulp was necessary in teeth of people of advanced years, where the pulp was almost entirely obliterated, although sensibility remained.

I have taken a spear-point drill, grinding same to a long taper, with which I have drilled into the tooth in the direction of the pulp as deeply as the patient would allow; then by forcing into this hole, having a gradual taper at the bottom, the square-end needle, as before described, an almost water-tight joint is formed which will allow great pressure to be made, whereby the cocaine solution will be forced into the fibrilla of the tooth, producing anæsthesia. You cannot force the solution to any great depth into the tissues, but far enough to drill deeper and repeat the operation. By this method I have been enabled to reach the apex of several teeth with little or no pain.

*Dr. Frank Gardiner.*—My experience in removing pulps with cocaine has been almost identical with that of Dr. Darby. I think it has been considerably over a year since I had to resort to the single application of arsenic. I have had one or two failures, and have had to treat the pulp by making applications; but I have not had to resort to arsenic. I fully endorse the idea of reaming the canal. I think that is a very strong point, and I also resort to the same method in the treatment of putrescent canals and in immediate root-filling. Of course, I would not fill immediately all putrescent canals with large openings at the apex. I do not think that it would be safe, owing to the danger of getting a slight quantity of septic material through the apex; neither do I always immediately fill a canal when extensive hemorrhage results from the removal of a live pulp. In all other cases I feel entirely safe in filling the canal at the first sitting, and have had more satisfaction and comfort from this method than I have from all the other methods combined.

*Dr. Wilson Zerfing.*—I would ask whether one can get anæsthesia of the pulp with cocaine-pressure after arsenic has been sealed in. I have repeatedly tried it with varying success, and very often with perfect success, in all stages of inflammation due to exposure. Aching pulps have come in, and I have introduced cocaine-pressure and extracted the pulp.

*The President.*—In using the pressure method the question arises whether the cocaine is forced into the substance of the pulp or around it, between the pulp and the pulp-chamber.

*Dr. Darby.*—If you put on pressure enough, it goes everywhere.

*Dr. M. J. Schamberg.*—I believe it is a very pertinent question whether anæsthesia is due mainly to the cocaine or to pressure. We know that both will produce anæsthesia, that cocaine if injected into tissue will produce a state of insensibility to pain, and we also know that by the so-called Schleich's method of infiltration anæsthesia, if we inject water or normal salt solution we can likewise produce insensibility to pain; and while I do not doubt that both are factors in the production of anæsthesia of the pulp, at the same time it is a point worthy of question which is the greatest factor, because I believe that we could then probably solve the reason why, when a pulp is partially removed by cocaine and pressure, you cannot remove the remaining pulp at a subsequent sitting by the same method, because there is not sufficient pulp there to fill the canal and you cannot exert the proper amount of pressure on it. By pressure upon it you force out most of the blood, and, at the same time, cause extreme pressure on the nerve-filaments. Then the question arises as to whether the anæsthesia is as profound in the teeth with large apical foramina as in those with small ones for the same reason. It was intimated that anæsthesia in the pulp of wisdom-teeth is not as successful as in other teeth and the same in molar teeth. I believe that it is largely due to the fact that you cannot exert uniform pressure on the entire pulp. I would like to hear an expression of opinion in regard to this point. I have had no experience in this direction, and merely speak of it in a more or less theoretical way, using my experience with local anæsthesia for surgical procedures; but I would like to ask whether the hemorrhage is greater in teeth in which the sensibility is not entirely destroyed than in those in which the anæsthesia has been profound.

*Dr. Gardiner.*—In my experience I am inclined to think that the hemorrhage proceeds from teeth with large foramina, owing to the attachment of the outside tissues and the size of the blood-vessels entering the canal. The hemorrhage did not come from the sensitive pulps. I would also say that I often had to repeat the application of cocaine, not only to those originally in a perfectly live condition, but also to those that were partly dead or where some portion of the pulp was putrescent and the remainder alive. These filaments sometimes are stubborn, but with repeated application I have been able to remove them all finally.

Subject passed.

*Dr. J. T. Lippincott.*—Perhaps the members of the Academy may be interested in a case of response to temperature in a tooth from which the pulp has been removed. The patient, a man perhaps forty-five years of age, had a superior first molar in which there had been a gutta-percha filling for a number of years, which I removed, and beneath it found a putrescent pulp. The roots were thoroughly cleansed and dressed. The dressing remained there probably from two to three months. The man was away from the city, and did not immediately come back on his return. When I next opened into that tooth I found the root-canals in apparently perfectly sterile condition; the canals and cavity were filled. Within perhaps two or three weeks the patient complained of a sensitiveness to heat and cold. He complained that he was not able to take a spoonful of soup, cup of coffee, or drink of ice-water without extreme pain which lasted anywhere from half an hour to an hour, and these paroxysms of pain were brought about not only by the contact with heat and cold in the mouth, but also when he went out in the cold. The tooth responded to applications to any point on its surface, but the most sensitive point was upon the distal surface at the gum margin. It failed to respond to ordinary applications for the relief of sensitive surfaces at that point, and upon the suggestion of Dr. Darby I applied the direct cautery, with, at first, but little apparent result, but after perhaps three applications the extreme sensitiveness disappeared and the tooth has become comfortable.

*Dr. Darby.*—That was one of the most interesting cases I ever saw. Dr. Lippincott brought his patient to my office. I began to question a little whether or not he erred in his diagnosis. The tooth had every appearance of a vital tooth. There was no opacity or discoloration, yet the tooth was so isolated that it could not be mistaken; the gentleman himself could not mistake pain in another tooth for pain in that one; neither could the test applications made come in contact with any other tooth. I explored the tooth all around with a probe, and finally it touched one point on the distal surface; the man winced and gave indication of pain. I touched it again, and he winced again. While we are not in the habit of finding devitalized teeth sensitive, even in their pericemental covering, yet we did find this sensitive spot there, and the man showed indications of pain every time I touched it. I suggested to Dr. Lippincott to use the galvano-cautery, and I am glad to know the result. The question is, What made that tooth sensitive?

*Dr. James Truman.*—It must have been due to the inflammation of the pericemental border. I had a number of cases where it was so hypersensitive,—indeed, I had one in my own mouth, and I had reason to know, therefore, the suffering it occasioned. It was quite as sensitive if touched with cold or heat. I have not used the actual cautery, which is very good; silver nitrate has generally done the work for me.

*Dr. Darby.*—This spot was an eighth of an inch below the gum line in that tooth.

*Dr. Truman.*—The pericementum is alive.

*Dr. Darby.*—This was not a recent resorption; it was an old one.

*Dr. Truman.*—At the point where the resorption ceases you have live pericementum.

*Dr. Darby.*—It could not be anything else with pulpless teeth.

*Dr. Louis Jack.*—Are you certain the pulp was entirely dead in the distal buccal root?

*Dr. Lippincott.*—I filled all three roots.

*Dr. Huey.*—How far did you go into those roots?

*Dr. Lippincott.*—I opened them up pretty thoroughly, though I did not carry my drill to the end of the buccal root, as I have heard some men say they do.

*Dr. Jack.*—I have heard of repeated instances where the upper molar was giving trouble, where the pulp was not devitalized at the end. I have had several instances; I had one in my own mouth, from which I suffered a great deal, although the endeavor had been made to devitalize entirely the pulp and fill a portion of the root, but it was not completely filled. I thought that the root disturbed me through reflex action.

*Dr. Darby.*—Was that tooth sensitive to cold or heat?

*Dr. Jack.*—I do not remember that.

*Dr. Darby.*—It was only sensitive to the touch, not so much to the changes, was it?

*Dr. Lippincott.*—In my case the tooth was extremely sensitive to thermal changes, to the application, for instance, of hot gutta-percha made on the buccal surface, besides the extremely sensitive point on the distal surface.

*Dr. Roberts.*—In that same connection with a devitalized tooth giving pain, I had an experience with a lower second molar rather different from that had by Dr. Lippincott. The tooth had given a good bit of trouble; I had tried to cap the pulp and keep it comfortable, but I finally took the filling out and went into it with cocaine. I filled both roots, and thought my trouble was over, but it was not more comfortable than before. I worked over it for two months, and then took the filling out of the roots, but could find nothing wrong, so again filled up the tooth. The patient went off comfortable, and has remained so. Now, what caused this I cannot say.

Adjourned.

Otto E. Inglis,
*Editor Academy of Stomatology.*

